# Impaired macrophage autophagy induces systemic insulin resistance in obesity

**DOI:** 10.18632/oncotarget.9590

**Published:** 2016-05-25

**Authors:** Young-Ho Kang, Mi-Hyang Cho, Ji-Young Kim, Min-Seo Kwon, Jong-Jin Peak, Sang-Wook Kang, Seung-Yong Yoon, Youngsup Song

**Affiliations:** ^1^ Department of Biomedical Sciences, University of Ulsan College of Medicine, Asan Institute for Life Sciences, Asan Medical Center, Seoul, Korea; ^2^ Alzheimer's Disease Experts Laboratory (ADEL), Department of Brain Science University of Ulsan College of Medicine, Asan Institute for Life Sciences, Asan Medical Center, Seoul, Korea; ^3^ Bio-Medical Institute of Technology (BMIT), University of Ulsan, College of Medicine, Seoul, Korea

**Keywords:** autophagy, adipose tissue macrophage, insulin resistance, obesity, reactive oxygen species, Pathology Section

## Abstract

Obesity-induced insulin resistance and diabetes are significantly associated with infiltrates of inflammatory cells in adipose tissue. Previous studies recognized the involvement of autophagy in the regulation of metabolism in multiple tissues, including β-cells, hepatocytes, myocytes, and adipocytes. However, despite the importance of macrophages in obesity-induced insulin resistance, the role of macrophage autophagy in regulating insulin sensitivity is seldom addressed. In the present study, we show that macrophage autophagy is important for the regulation of systemic insulin sensitivity. We found that macrophage autophagy is downregulated by both acute and chronic inflammatory stimuli, and blockade of autophagy significantly increased accumulation of reactive oxygen species (ROS) in macrophages. Macrophage-specific *Atg7* knockout mice displayed a shift in the proportion to pro-inflammatory M1 macrophages and impairment of insulin sensitivity and glucose homeostasis under high-fat diet conditions. Furthermore, inhibition of ROS in macrophages with antioxidant recovered adipocyte insulin sensitivity. Our results provide evidence of the underlying mechanism of how macrophage autophagy regulates inflammation and insulin sensitivity. We anticipate our findings will serve as a basis for development of therapeutics for inflammatory diseases, including diabetes.

## INTRODUCTION

Type 2 diabetes mellitus caused by insulin resistance combined with β-cell dysfunction is a complex metabolic disorder highly associated with cardiovascular diseases, blindness, and renal failure. Insulin resistance appears to be induced mainly by obesity, as approximately 85% of type 2 diabetes patients are obese [1]. Obesity is characterized by chronic low-grade inflammation and increased infiltration of proinflammatory (M1-like) macrophages into adipose tissue [2, 3]. Macrophages are a major source of inflammatory mediators such as interleukin-1β (IL1β), interleukin-6 (IL6), and TNFα [2–4]. Increasing evidence suggests a central role for adipose tissue macrophages in obesity-associated systemic insulin resistance [5–7].

Oxidative stress seems to be an important executor of obesity-associated insulin resistance and type 2 diabetes [1, 8–10]. The mitochondria are an important source of cellular reactive oxygen species (ROS). Mitochondrial dysfunction causes ROS accumulation and activates the nucleotide-binding domain, leucine-rich-containing family, pyd domain-containing-3 (NLRP3) inflammasome and caspase-1 [11–15]. Obesity induces mitochondrial dysfunction and accumulation of ROS in humans and mice [1, 16]. The attenuation of mitochondrial ROS generation in obese mice preserves glucose tolerance and insulin sensitivity [8].

Autophagy is an evolutionarily conserved catabolic process that sequesters cytoplasm, including aberrant organelles and macromolecules, into double-membrane vesicles for delivery to lysosomes for degradation and eventual recycling of the macromolecules [17]. Autophagy is involved in the pathogenesis of various human diseases, including metabolic syndromes [18–22]. Several studies have shown that insulin resistance and metabolic syndrome are distinctly influenced by autophagy in various tissues such as adipose tissues, skeletal muscles, pancreas, liver, and brain [23–30]. Autophagy in adipocytes regulates lipid accumulation in the body by controlling its differentiation and determines the balance between white and brown fat [29]. Autophagy in pancreatic β-cells is important for islet homeostasis and glucose homeostasis [27, 28]. Autophagy deficiency in skeletal muscles protects against insulin resistance via fibroblast growth factor-21 [26]. Autophagy defects in hepatocytes promote obesity-induced endoplasmic reticulum stress and provoke insulin resistance [31].

In macrophages, autophagy has been shown to be involved in controlling inflammation by regulating mitochondria turnover and ROS generation [14, 15]. However, despite the importance of macrophages in obesity and insulin resistance, the role of macrophage autophagy in regulating insulin sensitivity and glucose homeostasis is rarely addressed. In this study, we speculated whether autophagy in macrophages may regulate obesity-induced inflammation and insulin resistance.

## RESULTS

### Autophagy is downregulated in macrophages during inflammation

**Figure 1:**
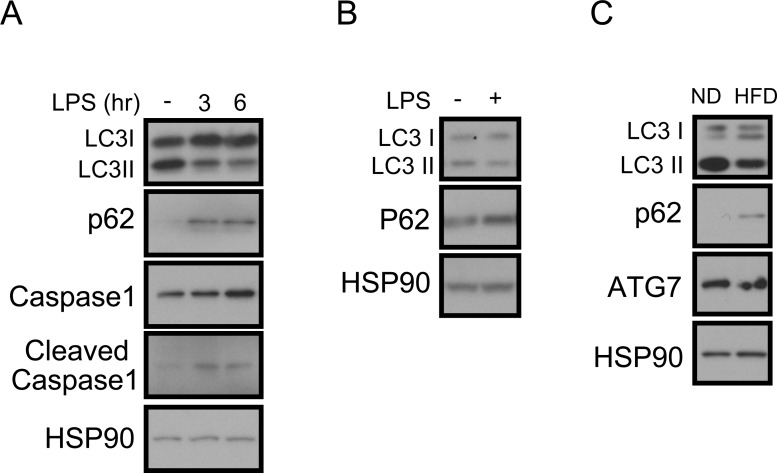
Impaired autophagy and increased IL1β in LPS-treated macrophages **A.** Western blots showing that LC3-II is decreased, p62-sequestosome-1 is increased, and the cleaved form of caspase-1 is increased in LPS (1 ng/mL)-treated BMDMs from C57BL6 mice. HSP90 was loaded as an internal control. **B.** Western blots showing that LC3-II is decreased and p62 is increased in the LPS-treated Raw264.7 macrophage cell line. **C.** Western blots showing that LC3-II conversion is decreased and p62 accumulation is increased in peritoneal macrophages from 60% HFD-fed obese mice compared with macrophages from normal chow diet (ND)-fed mice.

To study the role of autophagy in macrophages, we speculated whether this process was regulated by inflammatory stimulation. Treating bone-marrow-derived macrophages (BMDMs) derived from C57BL6 mice with lipopolysaccharide (LPS) appears to downregulate the autophagy process as observed by the decreasing LC3-II conversion and increasing the accumulation of p62, a marker of autophagic degradation (Figure 1A) [32]. As a reflection of inflammation status, caspase-1 cleavage increased and correspondingly IL1β secretion also increased (Figure 1A and Supplementary Figure 1A). A decrease in LC3-II conversion and an increase in p62 accumulation and IL1β secretion were similarly observed in LPS-treated Raw264.7 macrophage cells (Figure 1B and Supplementary Figure 1B). Because obesity is characterized by chronic low-grade inflammation, we wondered whether macrophage autophagy is downregulated in chronic inflammation. Peritoneal macrophages from mice fed a 60% high-fat diet (HFD) also showed downregulation of LC3-II conversion and upregulation of p62 compared with peritoneal macrophages from normal chow-fed mice (Figure 1C).

### Generation of macrophage-specific Atg7 knockout mice

Metabolic syndrome is often linked to chronic inflammation, and because autophagy is downregulated in macrophages by inflammatory stimuli (Figure 1), we wondered whether it plays a role in macrophages and whether there is a link between inflammation and insulin resistance. Accordingly, we generated a macrophage-specific autophagy knockout (KO) system by crossing Atg7*^fl/fl^* mice with LysMCre mice (Supplementary Figure 2A). Atg7 expression in BMDMs derived from Atg7*^fl/fl^*-LysMCre^+/−^ mice (referred to as Atg7KO mice hereafter) was decreased in comparison with control BMDMs obtained from LysMCre^+/−^ mice (referred to as control mice hereafter) by western blot and quantitative real time PCR (qRT-PCR) (Supplementary Figure 2B). As a reflection of the LysM expression in the myeloid lineage, the Atg7 expression levels in the liver and white adipose tissues were comparable, suggesting that the *Atg7* gene is specifically deleted in macrophages by LysMCre expression (Supplementary Figure 2C).

### Suppressing autophagy in macrophages is associated with insulin resistance without affecting obesity

At weaning, the Atg7KO mice appeared to weigh slightly more than the control mice, but the weights became comparable between these groups (Supplementary Figure 3A). The circulating glucose level of 5-h and 16-h fasted Atg7KO mice was also comparable to the control group. The glucose tolerance test (GTT) and insulin tolerance test (ITT) results also suggested that ATG7KO mice had a capability to regulate blood glucose levels comparable to the control mice (Supplementary Figure 3B-3C).

**Figure 2:**
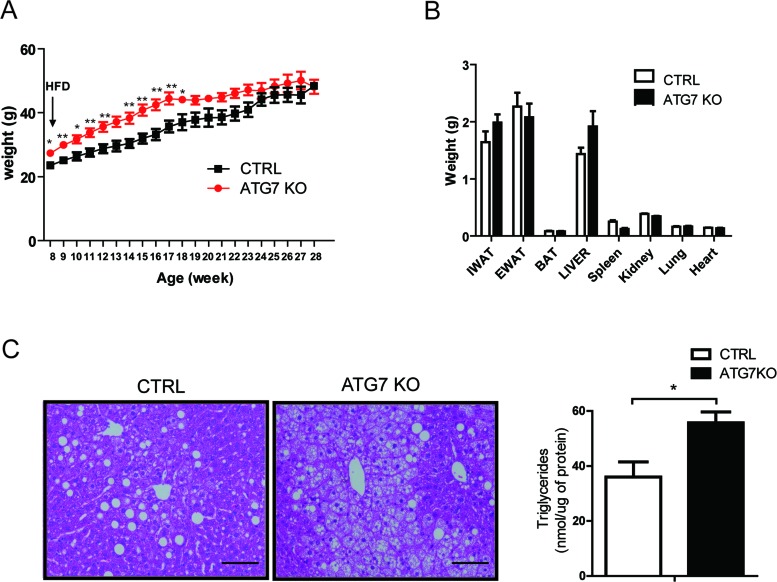
Macrophage-specific Atg7KO mice develop hepatic steatosis under HFD conditions **A.** Atg7^fl/fl^-LysMCre^+/−^ mice (Atg7KO) (*n* = 8) and LysMCre^+/−^ mice (control) (*n* = 6) were fed a 60% HFD, and their growth was monitored by weighing weekly. **B.** Twenty weeks after administering a 60% HFD, the tissues from Atg7KO (*n* = 8) and control mice (*n* = 6) were dissected and weighed. **C.** Liver sections from Atg7KO and control mice were prepared and stained with hematoxylin and eosin staining. Scale bar, 100 μm (left panel). The triglyceride content of liver tissues from control and Atg7KO mice is shown in the right panel.

We next challenged the ATG7KO mice with a 60% HFD. It appeared that the macrophage-specific deletion of the *Atg7* gene did not affect obesity. Initially, the HFD-fed Atg7KO mice weighed slightly more than the control mice, but the weights became comparable between the groups (Figure 2A). The metabolic cage studies showed that food intake, activity, and energy expenditure were also indistinguishable between the groups (Supplementary Figure 4A-4D). After 20 weeks of HFD feeding, we dissected various organs from the Atg7KO and control mice and measured their weights. Most organs including, the heart, kidney, lungs, and adipose tissue, had comparable weights in the Atg7KO and control groups, but the liver tissue weighed slightly more in the Atg7KO mice (Figure 2B). Interestingly, the liver tissues from the Atg7KO mice showed a severe accumulation of lipids in comparison with the control group (Figure 2C).

**Figure 3:**
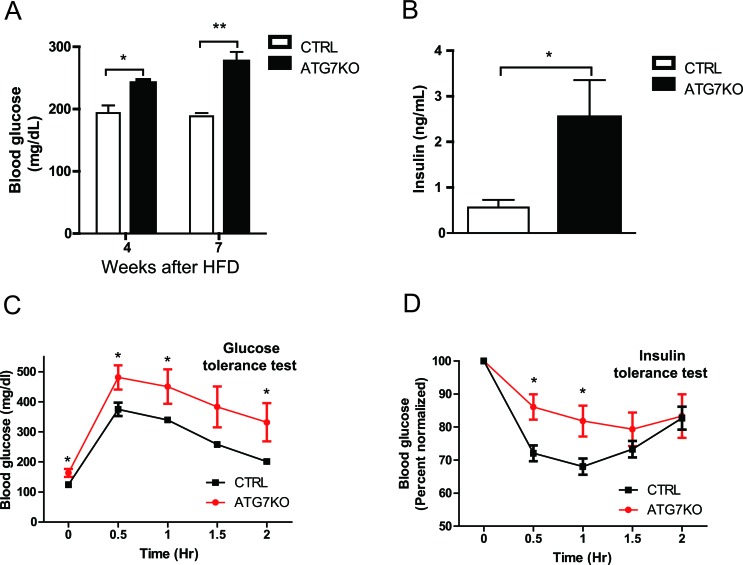
Impaired glucose homeostasis in macrophage-specific Atg7KO mice under diet-induced obesity conditions **A.** Circulating blood glucose levels in Atg7KO and control mice during a 60% HFD challenge. Circulating glucose levels were monitored in 5-h-fasted (weeks 4 and 7 after HFD) mice. **B.** Circulating insulin levels of Atg7KO (*n* = 6) and control (*n* = 6) mice were measured using ELISA. **C.** GTT and **D.** ITT (right) results for Atg7KO (*n* = 8) and control (*n* = 6) mice.

One of the major characteristics of insulin resistance is hepatic steatosis. Accordingly, the circulating glucose and insulin levels were found to be higher in Atg7KO mice (Figure 3A-3B), and correspondingly, the Atg7KO mice demonstrated a significantly lower respiratory exchange ratio (Supplementary Figure 4E) and impaired whole-body insulin sensitivity on the GTT and ITT (Figure 3C-3D).

### Elevated macrophage infiltration in the adipose tissue of Atg7KO mice

**Figure 4:**
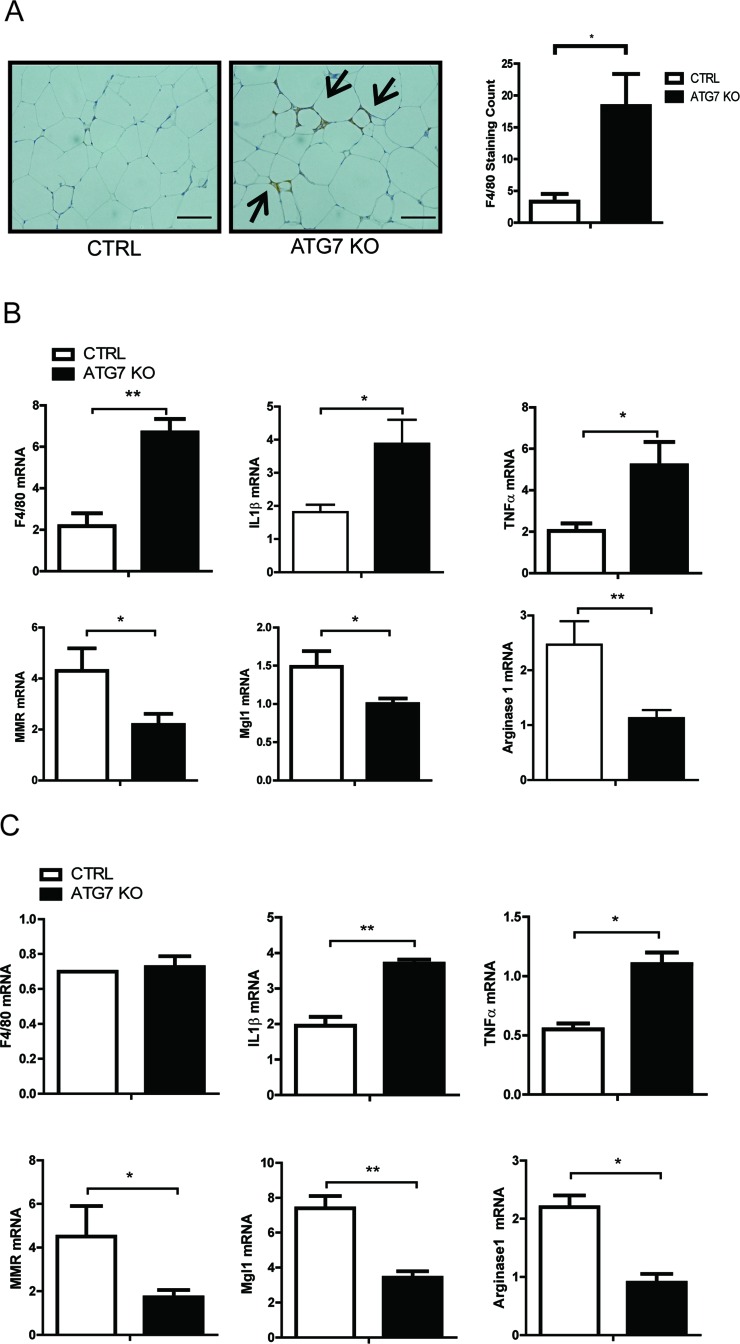
Increased proinflammatory macrophages in the adipose tissue of macrophage-specific Atg7KO mice **A.** Immunohistochemistry showing the increased infiltration of F4/80-positive macrophages in the adipose tissues of macrophage-specific Atg7KO mice. **B.** qPCR analysis showing that the F4/80, IL1β, and TNFα mRNA levels increased and the MMR, Mgl1, and arginase1 mRNA levels decreased in the adipose tissues of Atg7KO mice. **C.** qPCR analysis showing that IL1β and TNFα transcripts were upregulated and that MMR, Mgl1, and arginase1 mRNAs were downregulated in the peritoneal macrophages of Atg7KO mice.

Diet-induced obesity increases macrophage infiltration in adipose tissue and contributes to the development of systemic insulin resistance [2, 3, 33, 34]. We noticed that both the control and Atg7KO mice in our current study demonstrated increased macrophage accumulation in their adipose tissues, but this was higher in the Atg7KO mice (Figure 4A). Consistent with the immunostaining results, F4/80 mRNA expression in the adipose tissues of Atg7KO mice was increased, suggesting the systemic insulin resistance in these mice is associated with increased inflammatory infiltrates in the adipose tissues (Figure 4B). There are at least 2 types of adipose tissue macrophages that have been characterized, and it has been shown that the number of M1 macrophages and the M1/M2 macrophage ratio in adipose tissue are important determining factors for insulin sensitivity. When we analyzed the mRNA expression levels of the M1 and M2 macrophage markers, the M1 markers (including IL1β and TNFα mRNA) increased and the M2 markers (such as MMR, Mgl1, and agrinase1) decreased in the adipose tissue of Atg7KO mice (Figure 4B). Because we saw an increased number of M1 and decreased level of M2 macrophages in the adipose tissue, we hypothesized that autophagy might regulate the M1 and M2 macrophage polarization. To test this, peritoneal macrophages were collected from thioglycolate-injected control and Atg7KO mice, and the M1/M2 population was analyzed using qRT-PCR. Similar to adipose tissues, the IL1β-expressing and TNFα-expressing M1 macrophage population was found to be elevated in peritoneal macrophages from Atg7KO mice, and the M2 population of peritoneal macrophages, expressing MMR, Mgl1, and arginase1 were markedly decreased in Atg7KO mice (Figure 4C).

### Blocking autophagy increases ROS in macrophages

**Figure 5:**
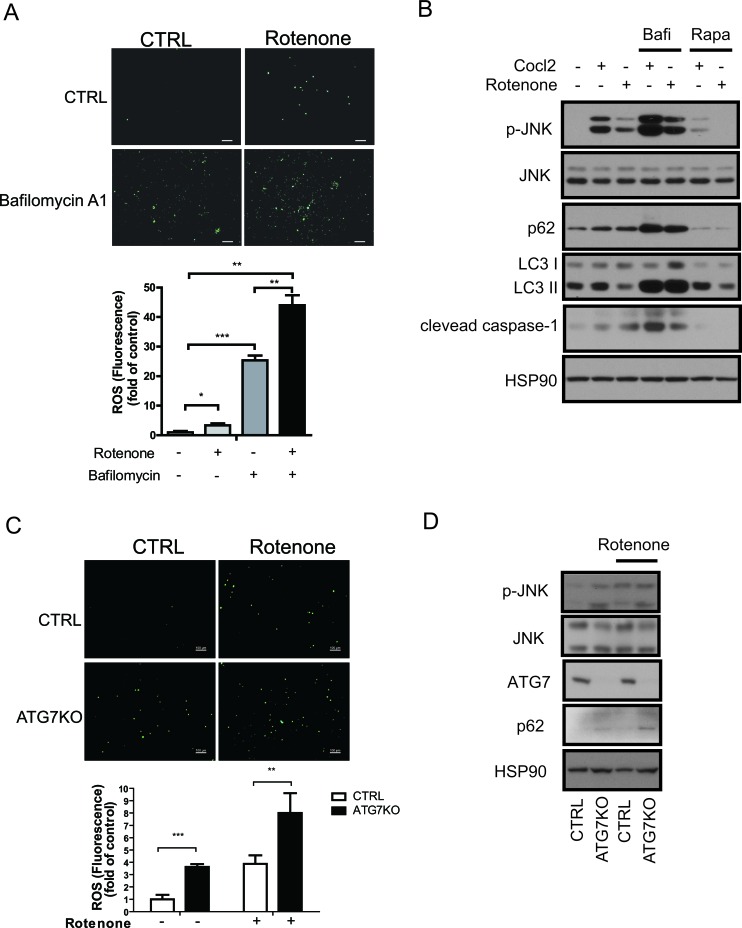
Increased ROS levels in autophagy-impaired macrophages **A.** ROS staining of Raw264.7 cells with DCF-DA dye revealed that the ROS levels increased in rotenone-treated or bafilomycin A1-treated cells. **B.** Western blots showing that phosphorylated JNK (p-JNK) and cleaved caspase-1 levels were increased in CoCl_2_-treated or rotenone-treated Raw264.7 cells and further increased in bafilomycin A1-treated cells and attenuated by rapamycin treatment. **C.** ROS staining of peritoneal macrophages with DCF-DA dye showing that the ROS level is increased in the Atg7KO mice and further increased by rotenone treatment. **D.** Western blots showing that the phosphorylation of JNK is elevated in the peritoneal macrophages of Atg7KO mice.

ROS induce inflammation, which in turn activates the NLRP3 inflammasome and interferes with insulin signaling [11]. Blocking autophagy disrupts mitochondrial homeostasis and elevates ROS levels [15, 35]. Accordingly, we found that rotenone treatments increased the ROS level in Raw264.7 cells, and an autophagy block with bafilomycin A1 further enhanced this increase (Figure 5A). It is well established that oxidative stress promotes c-Jun N-terminal kinase (JNK) activation [36, 37]. Consistent with the increase in ROS levels, CoCl_2_ and rotenone induced the phosphorylation of JNK, and bafilomycin A1 treatment further increased and rapamycin attenuated the cleavage of caspase-1 and JNK phosphorylation (Figure 5B). To determine whether the ROS level was increased in Atg7KO macrophages, peritoneal macrophages were isolated from Atg7KO and control mice and the ROS level was examined. As shown in Figure 5C and 5D, the ROS level and JNK phosphorylation in peritoneal macrophages were increased by rotenone treatments, and peritoneal macrophages from Atg7KO mice maintained a higher basal ROS level and JNK phosphorylation in comparison with peritoneal macrophages obtained from control mice. IL1β secretion was correlated with the ROS level. Treatment of Raw264.7 cells with rotenone stimulated IL1β secretion, and the conditioned media obtained from peritoneal macrophages of Atg7KO mice contained higher levels of IL1β and IL18. These levels were further boosted by rotenone treatment (Supplementary Figure 5A-C). Elevated TNFα mRNA level in peritoneal macrophages from Atg7KO mice (Figure 4C) and increased TNFα secretion was also observed (Supplementary Figure 5D).

### An impaired autophagy process disrupts insulin signaling in adipocytes

**Figure 6:**
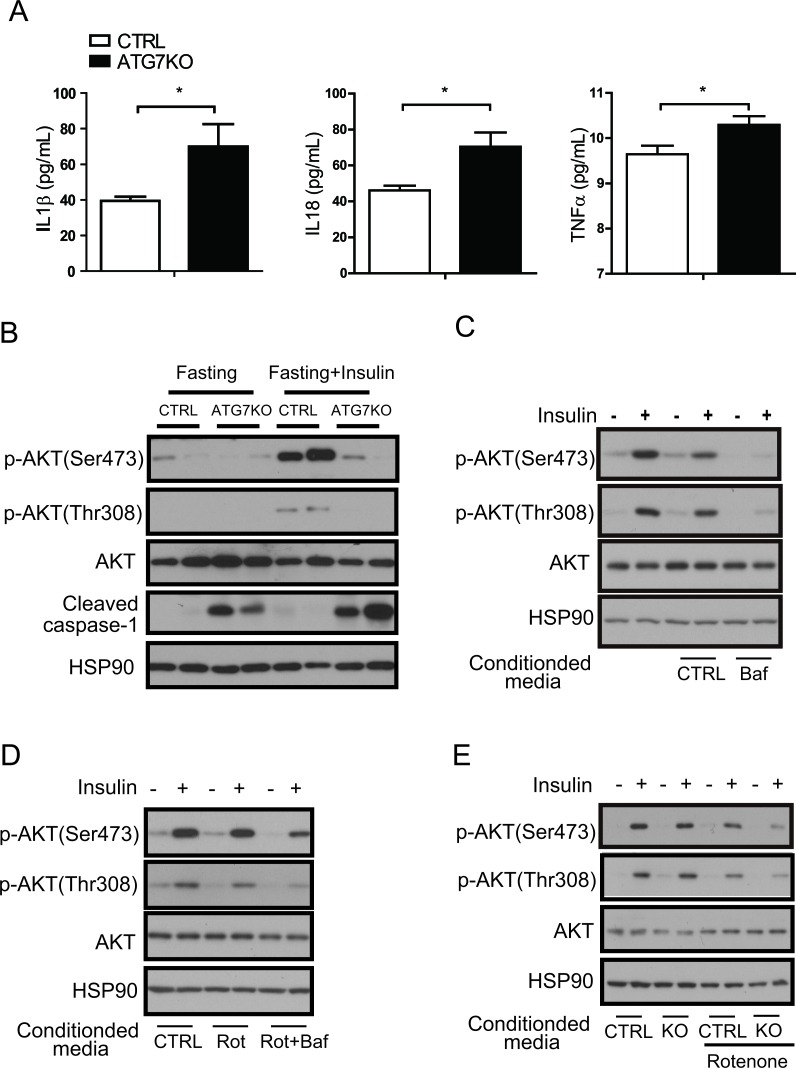
A macrophage autophagy block disrupts insulin signaling in adipose tissues **A.** Circulating IL1β, IL18, and TNFα levels in Atg7KO (*n* = 6-8) and control mice (*n* = 6-7) were examined by Elisa. **B.** White adipose tissue was collected after fasting (with or without insulin injection) from Atg7KO and control mice, and cleaved caspase-1 and insulin signaling were analyzed by western blot. **C.** Conditioned media collected from Raw264.7 cells treated with bafilomycin A1 and **D.** conditioned media collected from Raw264.7 cells treated with rotenone (or rotenone and bafilomycinA1) were applied to 3T3-L1 cells with or without insulin, and insulin signaling was then examined by western blot analysis. **E.** Conditioned media were collected from the peritoneal macrophages of the control and Atg7KO mice and applied to the 3T3-L1 cell line. The insulin signaling pathway was then examined by western blot analysis.

To verify whether insulin resistance is directly caused by impaired autophagy function in macrophages, we first examined insulin signaling in Atg7KO mice. The cleaved form of caspase-1 was increased in the adipose tissue of Atg7KO mice, in which elevated circulating IL1β, IL18, and TNFα levels were also observed (Figure 6A-6B). As expected, 16-h fasting downregulated AKT phosphorylation in the white adipose tissue and liver, and 16-h fasting followed by an insulin injection boosted AKT phosphorylation in both groups, however this phosphorylation was markedly attenuated in Atg7KO mice (Figure 6B and Supplementary Figure 6A).

ROS have been shown to stimulate inflammatory cytokine production in macrophages. Because we observed that the ROS level in Atg7KO macrophages increased, we tested whether the secreted cytokines from Atg7KO macrophages were the reason for the insulin resistance in these animals. We collected conditioned media from Raw264.7 cells that were treated with bafilomycin A1 and applied it to 3T3-L1 cells with or without insulin. As shown in Figure 6C, insulin elevated AKT phosphorylation, whereas conditioned media collected from bafilomycin A1-treated Raw264.7 cells inhibited this phosphorylation. Conditioned media collected from Raw264.7 cells that were treated with rotenone also downregulated AKT phosphorylation. Raw264.7 cells treated with bafilomycin A1 and rotenone demonstrated stronger effects, thereby suggesting that an autophagy block plays an additive or additional role in macrophages for the regulation of insulin signaling in 3T3-La preadipocytes and primary hepatocytes (Figure 6D and Supplementary Figure 6B). In accordance with these Raw264.7 data, the conditioned media from the peritoneal macrophages of Atg7KO mice more potently inhibited insulin-mediated AKT phosphorylation in 3T3-L1 preadipocytes and primary hepatocytes (Figure 6E and Supplementary Figure 6C).

**Figure 7:**
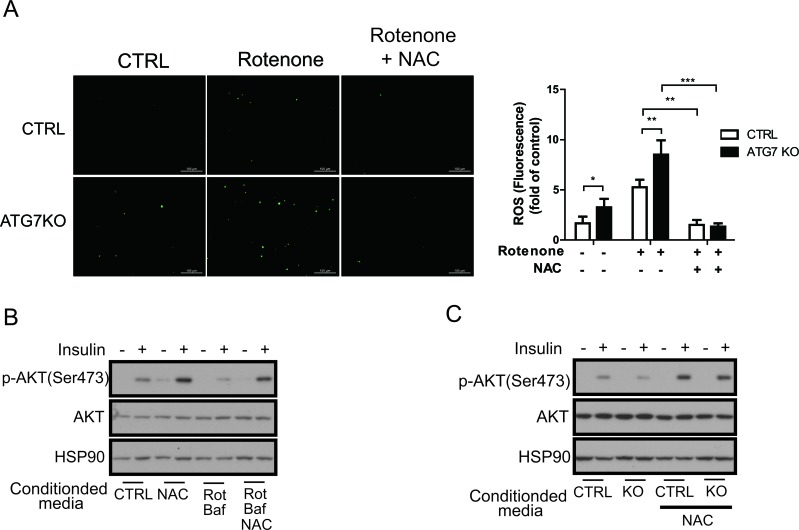
Macrophage ROS are required for the regulation of insulin signaling **A.** Peritoneal macrophages from control and Atg7KO mice were treated with rotenone with or without NAC. ROS level measured by DCF-DA staining shows that rotenone-mediated ROS generation is inhibited in peritoneal macrophages from both control and Atg7KO mice. **B.** Conditioned media were collected from Raw264.7 cells treated with NAC, rotenone, or bafilomycin A1 as indicated and applied to 3T3-L1 cells with or without insulin. Insulin signaling was examined by western blot analysis. **C.** Conditioned media collected from peritoneal macrophages of control and Atg7KO mice treated with NAC as indicated were applied to 3T3-L1 cells with or without insulin treatment, and insulin signaling was examined by western blot analysis.

To further examine the association between macrophage ROS and insulin resistance, we inhibited ROS generation in macrophages by treating with *N*-acetyl-l-cysteine (NAC). NAC treatments attenuated rotenone-mediated ROS generation in peritoneal macrophages derived from both control and Atg7KO mice (Figure 7A). Conditioned media collected from Raw264.7 cells treated with rotenone and NAC recovered insulin-induced phosphorylation of AKT compared with conditioned media collected from Raw264.7 cells treated with only rotenone (Figure 7B). Finally, conditioned media collected from Atg7KO-derived peritoneal macrophages, which were cotreated with rotenone and NAC, also recovered insulin-induced AKT phosphorylation compared with conditioned media treated with only rotenone (Figure 7C).

## DISCUSSION

We show for the first time that macrophage-specific Atg7KO mice exhibit impaired glucose tolerance and insulin sensitivity because of increased M1 macrophages, decreased M2 macrophages, and accumulation of ROS when receiving a HFD. Hence, autophagy in macrophages plays an important role in regulating systemic insulin sensitivity and glucose tolerance.

It appears that autophagy is impaired in chronic obesity. Atg7 is degraded by calcium-dependent protease calpain-2 [38], which is upregulated in an obese liver [31]. Administering a HFD for 16 weeks decreases Atg7 expression in the liver [31]. Chronic lipid exposure also alters the lipid composition of membranes, which decreases the fusion of autophagosomes and lysosomes and thereby results in impaired autophagy [39]. Inflammation is associated with obesity-associated insulin resistance [5, 6], and in our current experiments, treating macrophages with inflammatory cytokines impaired autophagy (Figure 1), further supporting the idea that autophagy in macrophages is impaired under obesity-induced inflammatory conditions.

Autophagy in different tissues and cells plays different roles in regulating glucose homeostasis and insulin sensitivity. This may be because autophagy has different effects on the characteristics of particular cells [25–29]. Our present study has revealed that autophagy in macrophages plays a significant role in regulating insulin sensitivity and glucose homeostasis (Figures 3 and 6). Insulin signaling is attenuated in the liver and adipose tissues of Atg7KO mice (Figure 6 and Supplementary Figure 6). Insulin signaling in adipocytes and hepatocytes is attenuated after treatment with conditioned media from autophagy-impaired macrophages (Figure 6 and Supplementary Figure 6).

Macrophages are classified as classically activated (M1) or alternatively activated (M2). M1 macrophages arise from stimulation by T helper type 1 (Th1) cytokines (IFNγ, TNFα, and LPS) and secrete proinflammatory cytokines (TNFα and IL1β/6/12/18). M2 macrophages arise from stimulation by T helper type 2 (Th2) cytokines (IL4/13) and highly express the mannose receptor and secrete various anti-inflammatory molecules (YM1, IL10, and Fizz-1). In atherosclerotic plaques, autophagy-deficient macrophages demonstrate inflammasome activation, increased IL1β production, and the accumulation of cholesterol crystals. Hence, autophagy is necessary for the clearance of cholesterol and the regulation of IL1β production in macrophages by promoting the M2 phenotype [40, 41]. Inhibition of autophagy processes via a lysosomal inhibitor, including chloroquine or bafilomycin A1, results in defects in M2 macrophage polarization [25]. The role of autophagy in regulating the M1/M2 population under HFD conditions is also supported by a recent report by Liu et al [42]. In this study, macrophage-specific Atg5KO mice demonstrated increased proinflammatory macrophage polarization under HFD conditions in combination with LPS treatment. Our current observation of a higher proportion of M1/M2 macrophages in the adipose tissue of Atg7KO mice (Figure 4) also suggests that autophagy in macrophages is important for regulating systemic insulin sensitivity and glucose tolerance by regulating the M1/M2 proportion in obesity.

ROS promote insulin resistance and is associated with obesity and type 2 diabetes mellitus [8–10]. Oxidative stress promotes JNK activation [36, 37]. Obesity-induced insulin resistance and inflammation is aggravated by JNK expression in macrophages [43]. Mitochondrial dysfunction causes ROS accumulation [11] and has been shown to be involved in NLRP3 inflammasome activation [12–15]. Activation of the NLRP3 inflammasome results in the proteolytic cleavage of procaspase-1 to produce mature caspase-1, which cleaves pro-IL1β and pro-IL18 and secretes IL1β and IL18 [32, 44]. In prediabetes and diabetes conditions, circulating levels of IL1β, IL18, and IL1RA, which are induced by IL1β, are elevated [19, 45], and treatment with IL1β induces insulin resistance in adipocytes, implying that IL1β and IL18 have a crucial role in the development of type 2 diabetes [46]. Autophagy in macrophages is important for preserving mitochondrial integrity regulating ROS generation, and activating NLRP3 inflammasomes [14, 15, 47]. Mitochondria are a rich source of cellular ROS. Elevation of ROS induces autophagy to eliminate damaged mitochondria and suppresses cellular ROS levels. Unsuccessful clearance of damaged mitochondria, for example, by preventing autophagosome formation, increases ROS levels. Our observation of higher ROS and phosphorylated JNK in the macrophages of Atg7KO mice (Figure 5) supports that autophagy in macrophages is important for regulating systemic insulin sensitivity and glucose tolerance via ROS, JNK, and IL1β signaling pathways. In fact, treating type 2 diabetes patients with anakinra, a recombinant IL1R antagonist, decreased inflammation and improved glycemia [18]. Canakinumab, a neutralizing antibody for IL1β, is under clinical trial for diabetes [16, 20].

Accumulating evidence suggests that oxidative stress along with excessive lipid accumulation in liver, adipose tissue, muscle, and pancreas is the leading cause of insulin resistance. These conditions are induced or exacerbated by overnutrition and are strongly associated with inflammation [21, 22]. For example, it has been shown that the NLRP3 inflammasome is activated by saturated free fatty acids [48] and is involved in obesity-induced inflammation and insulin resistance [49]. Accordingly, we found that Atg7KO mice displayed insulin resistance without accompanying body weight changes compared with control mice under HFD conditions. Activation of autophagy by rapamycin or resveratrol or by treating obese mice with apocynin, a ROS blocker, improved glucose homeostasis without weight loss [1, 19–22]. Hence, we hypothesize that autophagy impairment in macrophages may aggravate inflammation and ROS generation, leading to insulin resistance in Atg7KO mice, especially under HFD conditions.

**Figure 8:**
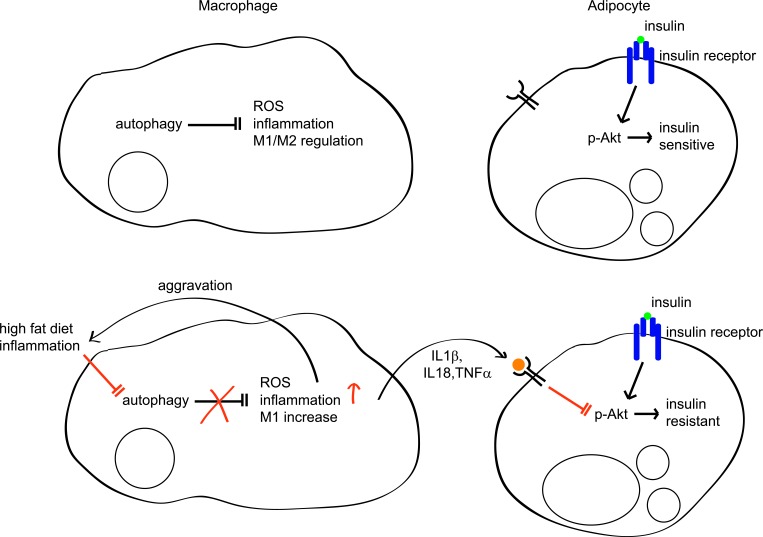
Regulation of insulin sensitivity by autophagy in macrophages In normal conditions, autophagy regulates inflammation, ROS levels, and M1/M2 populations in macrophages. In this context, adipocytes are sensitive to insulin. However, in inflammatory conditions such as diabetes or HFD, autophagy is impaired in macrophages and its regulation of ROS, inflammation, and the M1/M2 population is also impaired, which further aggravates inflammation and autophagy impairment. Concomitantly, increased inflammatory cytokines such as IL1β, IL18, and TNFα inhibit Akt, which impairs insulin signaling, resulting in insulin resistance.

In conclusion, autophagy in macrophages plays a significant role in maintaining insulin sensitivity and glucose homeostasis by regulating macrophage polarization and ROS generation. HFD-induced inflammatory conditions impair autophagy in macrophages, which may provoke production of ROS and proinflammatory M1 cytokines, further aggravating inflammation and autophagy inhibition, resulting in a vicious cycle (Figure 8). We suggest that enhancing macrophage autophagy may be a viable therapeutic or preventative approach to inflammatory disease, including obesity-induced insulin resistance and diabetes.

## MATERIALS AND METHODS

### Macrophage-specific Atg7KO mouse model

Previously described Atg7*^fl/fl^* mice [50] and LysMCre knockin mice, acquired from The Jackson Laboratory (Bar Harbor, ME, USA), were crossed to produce macrophage-specific Atg7 conditional KO mice, i.e., Atg7*^fl/fl^*-LysMCre^+/−^. Deletion of the *Atg7* gene was confirmed by genotyping, and its expression was examined by western blot and qRT-PCR. Mice were kept in temperature-controlled housing under a 12-h light/12-h dark cycle (lights on at 8:00 AM and off at 8:00 PM) with free access to water and a normal chow diet (Purina Rodent Chow, 38057). For the HFD study, the normal chow diet was switched to a 60% HFD (Research Diets, D12492) when the mice were 6-8 weeks of age. All animal studies were conducted according to an approved protocol from the Institutional Animal Care and Use Committee of Asan Life Science Institute, Asan Medical Center, Seoul, Korea.

### Cell culture and isolation of BMDMs and peritoneal macrophages

Raw264.7 and 3T3-L1 cell lines were cultured in Dulbecco's minimal essential medium (DMEM) supplemented with 10% FBS (Hyclone, Logan, UT, USA) and 1% penicillin/streptomycin. For BMDMs, bone-marrow cells were collected from the femur and tibia of mice and cultured in α-MEM supplemented with 10% FBS and 30 ng/mL macrophage colony-stimulating factor. Unattached cells were removed daily, and fresh culture media was replaced every 3-4 days. For peritoneal macrophage isolation, thioglycolate-stimulated macrophages were collected from Atg7*^fl/fl−^*LysMCre^+/−^ and control mice by peritoneal lavage 3-4 days after the intraperitoneal injection of sterile 3.85% thioglycolate. The isolated cells were washed with PBS and plated at a density of 106 cells/mL of DMEM/10% FBS/penicillin/streptomycin. The next day, the floating cells were washed away and the attached macrophages were treated and analyzed as indicated.

### Reagents and treatments

A final concentration of 1-10 ng/ml LPS (Sigma Chemical Co., St. Louis, MO, USA), 5-μM rotenone (Tocris, Bristol, UK), 0.5-μM CoCl_2_ (Sigma), 0.1-μM bafilomycin A1 (Tocris), 200-nM rapamycin (Tocris), and 20 mM NAC (Sigma) was applied to the cells as indicated in the figures. For the in vivo insulin signaling study, 2-U/kg Humulin (Lilly, Indianapolis, IN, USA) was intraperitoneally injected, and for the in vitro insulin signaling study a final concentration of 50 ng/mL insulin was administered.

### Metabolic cage studies

Metabolic cage studies were conducted as previously described [51]. Briefly, mice were individually housed for acclimation before the experimental day. O_2_ consumption, CO_2_ production, locomotor activity, and food intake were then monitored using an indirect calorimeter (Columbus Instruments, Columbus, OH, USA).

### GTT and ITT

For the GTT, 16-h fasted male mice were intraperitoneally injected with glucose (1.5-2 g/kg depending on the weight of mice), and glucose level was measured every 30 min. For the insulin tolerance test (ITT), the male mice fasted for 4-5 h, and 1-1.2-U/kg insulin (Humulin, Lilly) was intraperitoneally introduced. For both GTT and ITT, the glucose level was measured immediately after collecting blood from the tail vein using an Accu Chek Performa glucometer (Roche, Basel, Switzerland).

### Plasma analysis

The circulating insulin (Alpco, Salem, NH, USA) and IL1β, IL18, and TNFα levels were assessed using ELISA according to the manufacturer's guidelines (R&D Systems, Inc., Minneapolis, MN, USA).

Histology

Isolated tissues from mice were immediately fixed and embedded in paraffin. Sections (5-μm thick) were stained with hematoxylin. Eosin or immunohistochemical staining was performed following F4/80 antibody (Abcam, Cambridge, UK) incubation using the avidin-biotin complex method (Vector Laboratories, Burlingame, CA, USA).

### Sample preparation and protein analysis

Immediately after dissection, the mouse tissues were quickly frozen in liquid nitrogen and kept at −80°C until further processing. The tissue samples were ground in liquid nitrogen and lysed with RIPA buffer supplemented with a proteinase inhibitor (Roche) and phosphatase inhibitor. For immunoblotting analysis, antibodies for Atg7, p62, phospho-JNK, phospho-AKT (Thr308), phospho-AKT (Ser473) (Cell Signaling Technology, Danvers, MA, USA), JNK, caspase-1, heat shock protein 90 (Santa Cruz Biotechnology, Inc., Santa Cruz, CA, USA), and LC3 (Sigma) were used.

### RNA isolation and qRT-PCR

Total RNA was extracted using the Tri-RNA reagent or RNA mini kit (Favorgen, Taiwan) according to the manufacturer's instructions, and 500 ng of total RNA was used for the first cDNA synthesis (Toyobo, Osaka, Japan). The synthesized cDNA was diluted 10-fold, and mRNA expression was quantified using qRT-PCR.

### ROS measurements

The total cellular ROS level was analyzed using 2′,7′-dichlorofluorescein diacetate (DCF-DA) reagent. Briefly, the cells were treated with various drugs as indicated in the figures and replaced with 4-μM DCF-DA in PBS for 5-20 min. After washing with PBS, the ROS level was analyzed in terms of the fluorescence intensity at 485-nm excitation and 530-nm emission wavelengths.

### Statistics

Data is presented as the mean ± SEM, and statistical analysis was performed by unpaired Student's *t*-test using GraphPad Prism. In this study, *p* < 0.05, *p* < 0.01, *p* < 0.001 represent *, **, and *** respectively and were considered statistically significant.

## SUPPLEMENTARY MATERIAL FIGURES


