# Supplementary material for: Impaired macrophage autophagy induces systemic insulin resistance in obesity
Source: Oncotarget. 2016 May 25;7(24):35577–91. doi: 10.18632/oncotarget.9590 (PMC5094946; doi:10.18632/oncotarget.9590)
Supplement: Supplementary file 1 [file oncotarget-07-35577-s001.pdf]

Impaired macrophage autophagy induces systemic insulin resistance in obesity

Supplementary Material

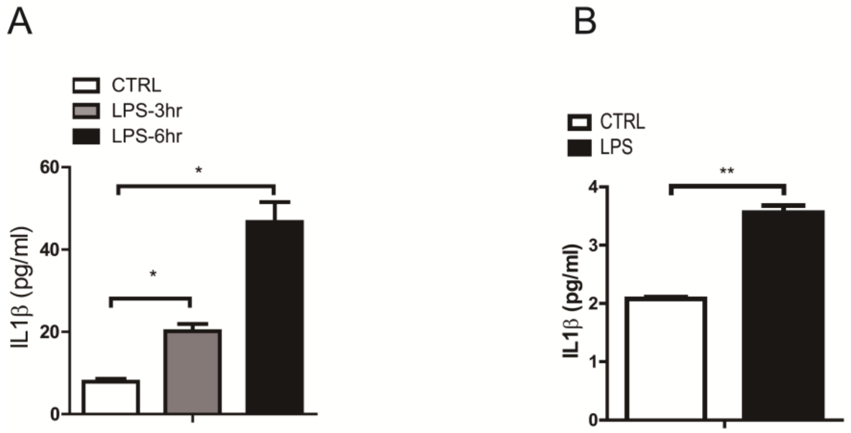

Supplementary Figure 1: Increased IL1β secretion in LPS-treated macrophages (A) ELISA analysis showing that IL1β secretion was increased in the LPS-treated BMDMs. **(B)** ELISA analysis showing increased IL1β secretion in the LPS-treated Raw264.7 macrophage cell line.

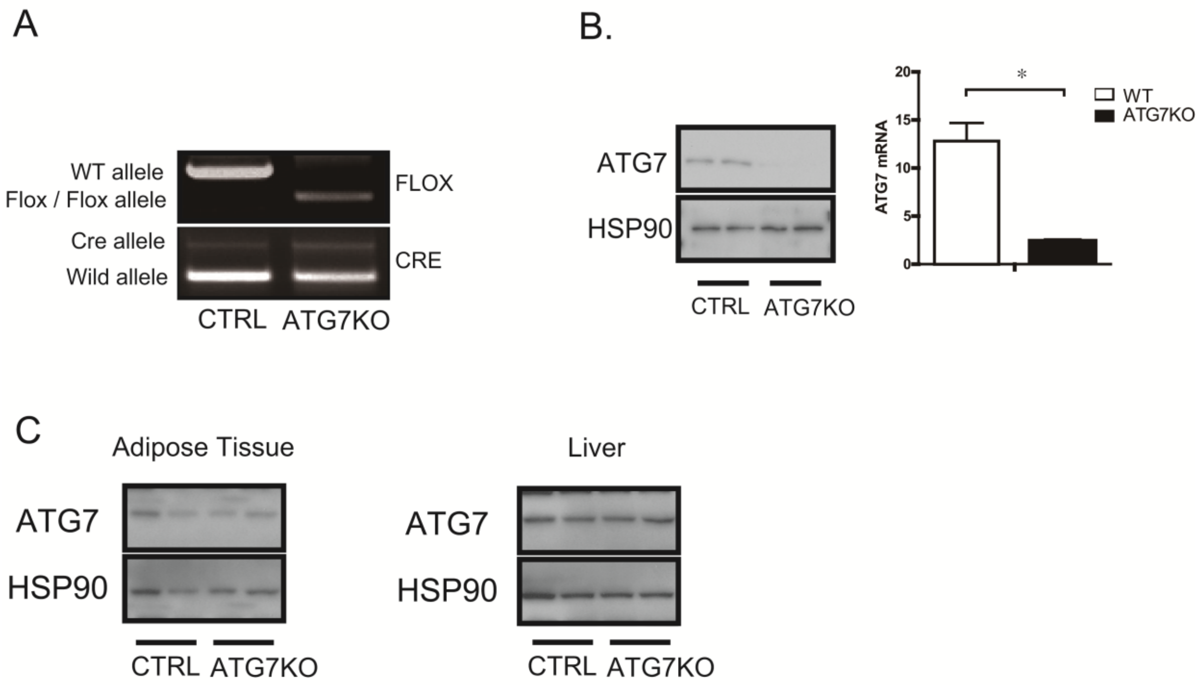

Supplementary Figure 2: Generation of macrophage-specific Atg7KO mice. **(A)** Genotyping results for Atg7$^{fl/fl}$-LysMCre$^{+/-}$ (Atg7KO) and LysMCre$^{+/-}$ mice (control mice). The top panel shows the genotyping results for the flox/flox allele, and the bottom panel shows the results for the Cre allele **(B)** Western blot (left) and qRT-PCR (right) analysis of Atg7 expression in BMDMs from Atg7KO and control mice showing that Atg7 protein expression was decreased in Atg7KO mice. **(C)** Western blot analysis of Atg7 expression in adipose tissue (left) and liver tissue (right).

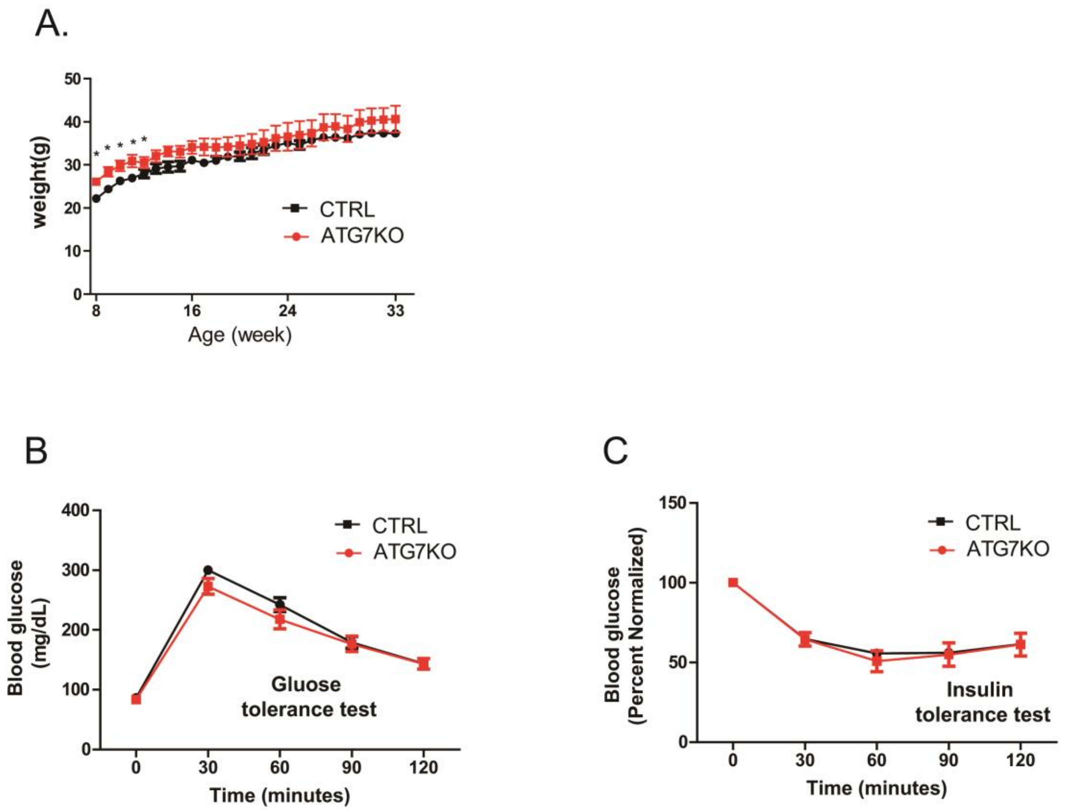

Supplementary Figure 3: Growth and glycemia in standard chow-fed macrophage-specific Atg7KO mice are comparable to the controls. (A) Growth curves, **(B)** GTT analysis, and **(C)** ITT analysis of standard chow-fed Atg7KO (n = 7) and control (n = 9) mice.

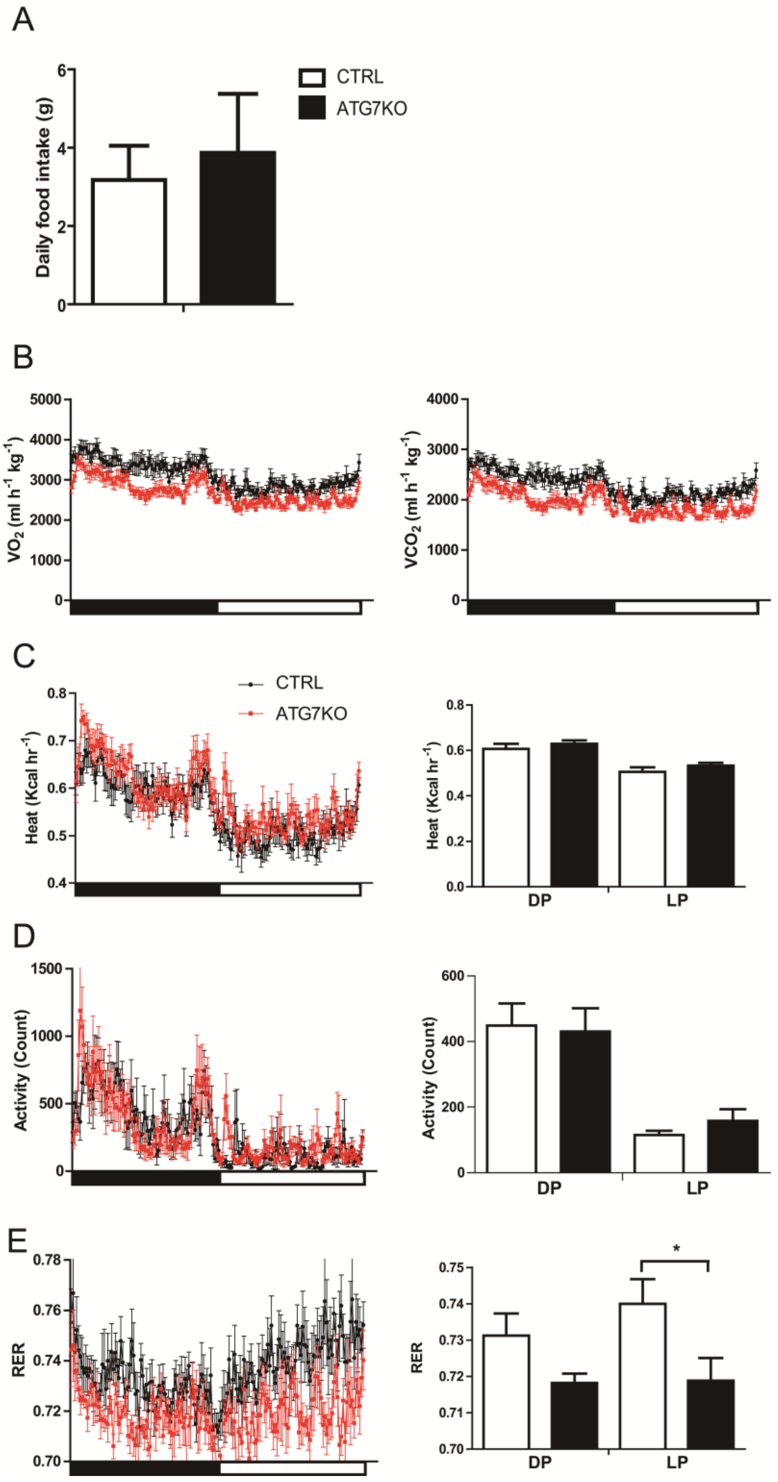

Supplementary Figure 4: Metabolic analysis of macrophage-specific Atg7KO mice. Atg7KO mice fed a 60% HFD (n = 3) and control mice (n = 4) were placed in metabolic cages, and their **(A)** food intake, **(B)** VO_2 and VCO_2, **(C)** energy expenditure, **(D)** activity, and **(E)** respiratory exchange ratios were measured.

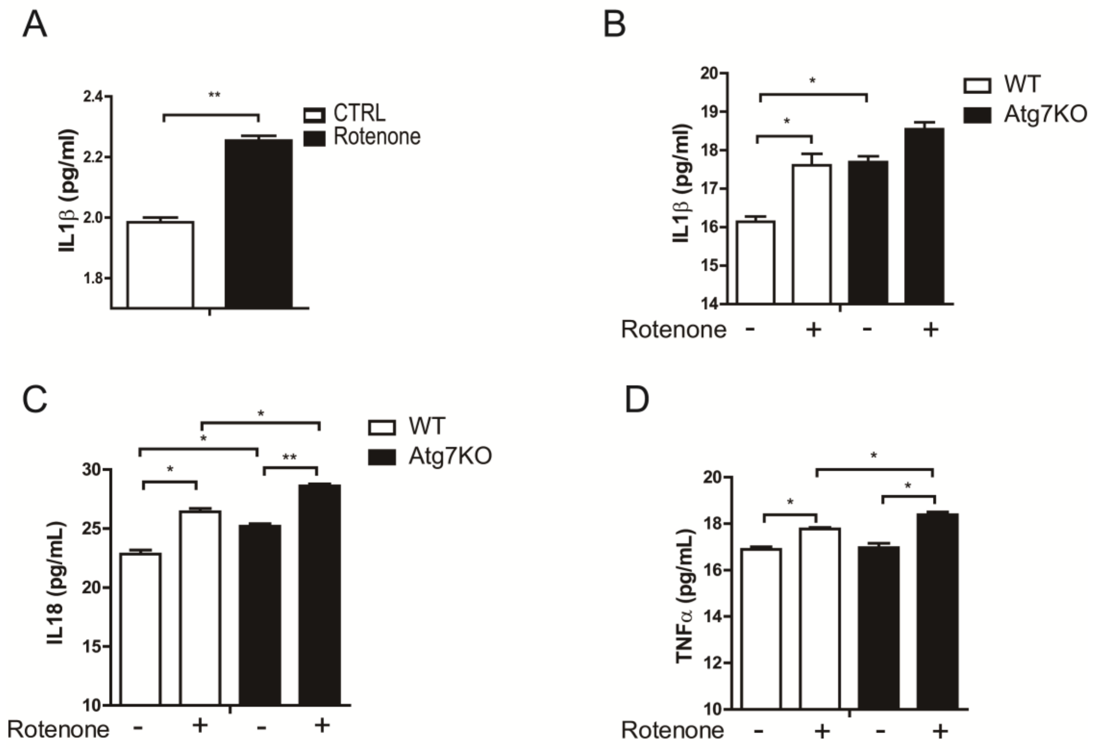

Supplementary Figure 5: ROS increases inflammatory cytokine secretion in macrophages. **(A)** IL1β secretion from Raw264.7 cells with or without rotenone treatment and **(B)** IL1β, **(C)** IL18, and **(D)** TNFα secretion from peritoneal macrophages obtained from control or Atg7KO mice with or without rotenone treatment.

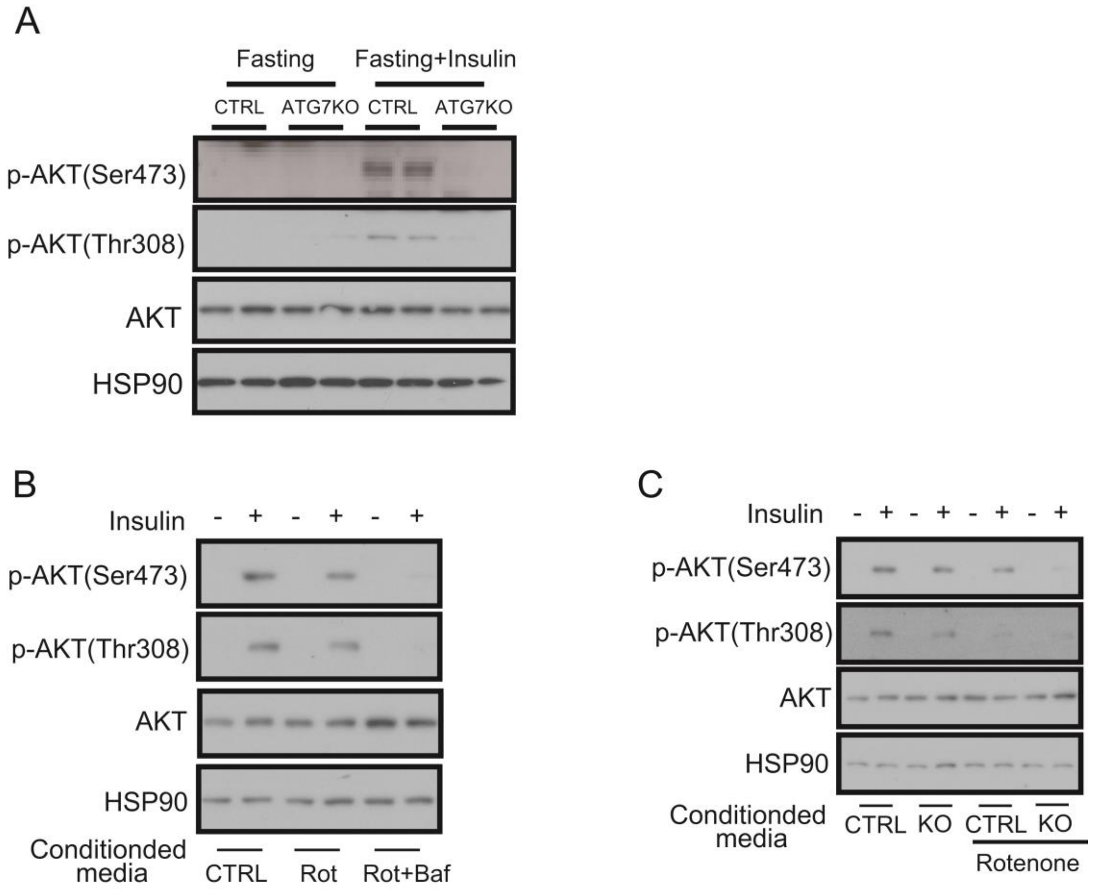

Supplementary Figure 6: Macrophage autophagy regulates insulin signaling in the liver and primary hepatocytes. (A) Insulin sensitivity was assessed by the AKT phosphorylation status in the liver tissues of Atg7KO and control mice. **(B)** Conditioned media collected from Raw264.7 cells treated with rotenone (or rotenone and bafilomycin A1) or **(C)** conditioned media collected from the peritoneal macrophages of the control and Atg7KO mice were applied to primary hepatocytes with or without insulin. Insulin signaling was then examined by western blot analysis.
